# Observations on James's Powder

**Published:** 1811-04

**Authors:** 


*To the Editors of the Medical and Physical Journal.*

Gentlemen,

I MET with the following Recipe for the preparation of James's Powder among some medical Extracts belonging to my late father; it had been in his possession near forty years. As it bears a great resemblance to the able analysis of James's Powder by Mons. Pully, in the *Annales de Chimie*, and inserted in the 8th volume of the Repertory of Arts, you will oblige me by inserting this along with the enclosed Recipe.

R. Reguli antimon.* sal. nitri. ana ponder. æqual. separatim in pulverem trita probe misceantur, deinde gradatim injiciatur mixtura in crucibulum; materiam ab igne remotam aqua bulliente ablua, et pulverem subtilissimum tere.

R. Pulv. ut sup. ℈ss. ad ℈j. mercur. D. 6ta. sublim. gr. 1 M. ft. pulv.

Perhaps no preparation in the whole range of pharmacy has given rise to more discussion than the preparation of this active mineral, yet we are still scarcely agreed as to the best mode of preparing it, or even as to its composition, and after all, perhaps, the method of oxydating antimony by the heating it in contact with nitre, though less free from impurities, may be considered as producing an oxyd, in which the extraneous matter is less important with respect to its employment in pharmacy.

Your's,

A. B.

---

* Vid. regulus antimon. James's Dispensatory and his succeeding Note.

 Experiment I.

### Experiment I.

I took (says M. Pully) nineteen decigrammes of James's Powder, and put them to infuse for some minutes in a small quantity of hot distilled water. I separated the water from them by the filtre, and I obtained by the evaporation of this liquid a salt, which had all the characters of the sulphate of potash. This salt, dissolved and treated with barytes, presented a precipitate of sulphate of barytes.

### Experiment II.

As I had perceived, before decomposing the salt by barytes, that the solution contained an excess of free potash, I wished to ascertain whether this potash did not hold in suspension a small quantity of oxyd of antimony. Having decanted the liquor in order to separate it from the sulphate of barytes, I poured into it sulphurated hydrogene, which immediately formed golden sulphur of antimony ; consequently the free potash was combined with a portion of antimony at the *minimum* of oxydation. Dr. Pearson speaks neither of this combination nor of the sulphate of potash.

### Experiment III.

I took the James's Powder which had been washed with hot distilled water, and heated it with nitric acid of 20 degrees. This acid dissolved the phosphate of lime without attacking the oxyd of antimony at the maximum. I separated this oxyd from the solution, and poured into the liquor ammoniac, which precipitated the phosphate of lime.

### Experiment IV.

I decomposed the phosphate of lime by weak sulphuric acid, and afterwards re-composed it by lime-water, in order to determine its proportions.

### Experiment V.

I took the oxyd of antimony at the *maximum* of oxydation, and dissolved it in muriatic acid. This solution, treated by sulphurated hydrogene, produced a hydrosulphuret of antimony, containing more sulphur than the Kermes mineral, and less than the sulphur auratum.

According to these experiments, of which I weighed all the products, the nineteen decigrammes of James's Powder which I analyzed were composed of

Decigrammes.

| | Decigrammes. |
|---|---|
| Oxyd of antimony, at the maximum . . . | 7 |
| Phosphate of lime . . . . | 4 |
| Sulphate of potash . . . | 4½ |
| Free potash, containing oxyd of antimony at the minimum . . . . . | 3½ |
| | 19 |

To recompose this powder, we must take,

| | |
|---|---|
| Sulphate of antimony . . . | 2 parts |
| Calcined phosphate of lime . | 1½ |
| Nitrate of potash . r . | 4 |

These substances are to be pulverised, mixed, and triturated. They are then to be put into a crucible, which is to be closed, and strongly heated. During this operation, the oxygene of the nitric acid, acting upon the sulphur of the sulphuret of antimony, converts it into sulphuric acid, which unites with a portion of the potash, and forms sulphate of potash; the rest of the free potash retains antimony oxydated at the minimum. The white powder which remains in the crucible is the same as that which is sold at such a high price by the English.

